# Indoor Air in Beauty Salons and Occupational Health Exposure of Cosmetologists to Chemical Substances

**DOI:** 10.3390/ijerph7010314

**Published:** 2010-01-26

**Authors:** Alexandra Tsigonia, Argyro Lagoudi, Stavroula Chandrinou, Athena Linos, Nikos Evlogias, Evangelos C. Alexopoulos

**Affiliations:** 1 Department of Aesthetics and Cosmetology, Technological Educational Institute of Athens, 12210 Athens, Greece; E-Mail: nikal12@hotmail.com (A.T. & N.E.); 2 Department of Hygiene and Epidemiology, School of Medicine, Athens University, 11527 Athens, Greece; E-Mail: alinos@med.uoa.gr; 3 Terra Nova L.t.d., Environmental Engineering Consultancy, 11527 Athens, Greece; E-Mails: lagoudi@terranova.gr (A.L.); chandrinou@terranova.gr (S.C.); 4 Occupational Health Unit, Department of Public Health, School of Medicine, Patras University, 26500 Rio Patras, Greece

**Keywords:** cosmetologists, beauty salons, nail salons, indoor air, occupational health, chemical exposure, volatile organic compounds, toluene, Greece

## Abstract

The indoor environment in four beauty salons located in Athens (Greece) was examined in order to investigate the occupational health exposure of cosmetologists to various chemical products typically used in their work. Chemical substances chosen for investigation were volatile organic compounds (VOCs), formaldehyde, ozone and carbon dioxide. Total VOCs levels measured showed significant variation (100–1,450 μg m^−3^) depending on the products used and the number of treatments carried out, as well as ventilation. The main VOCs found in the salons were aromatics (toluene, xylene), esters and ketones (ethyl acetate, acetone, *etc*.) which are used as solvents in various beauty products; terpenes (pinene, limonene, camphor, menthenol) which have a particular odor and others like camphor which have specific properties. Ozone concentrations measured in all salons were quite low (0.1 and 13.3 μg m^−3^) and formaldehyde concentrations detected were lower than the detection limit of the method in all salons (<0.05 ppm). Carbon dioxide levels ranged between 402 and 1,268 ppm, depending on the number of people present in the salons during measurements and ventilation. Cosmetologists may be exposed to high concentrations of a mixture of volatile organic compounds although these levels could be decreased significantly by following certain practices such as good ventilation of the areas, closing the packages of the beauty products when not in use and finally selecting safer beauty products without strong odor.

## 1. Introduction

Cosmetologists and beauticians and to some extent customers are exposed to high concentrations of several compounds that are included in the various chemical products used in their work/treatments. A wide diversity of chemical products are used in the different therapies performed in the beauty salons (facial cleansing, skin, nails and body hydrotherapy and care, anti-wrinkle, pigmentation and acne treatment, make up, depilation, body and face massage, reflexology, aromatherapy, face and body hair removal, *etc*.). Each of these products has a large number of components including several Volatile Organic Compounds (VOCs), methacrylates, phthalates, and formaldehyde. In addition, the use of various equipments can contribute to the increase of other chemical parameters such as ozone during the use of steam equipment (“ozonizer”), carbon monoxide during laser hair removal—photo-depilation, *etc*. The variations of chemical exposures have been described in a few studies mainly focused on hairdressing and nail salons [1–7].

Occupational skin and respiratory disorders, and disputable reproductive and genotoxic effects have been linked to chemical exposures of beauty workers [8–16]. Although industrial hygiene evaluations have found that airborne exposures are very low compared with occupational exposure standards and those found in industry, the levels of VOCs found in the indoor air can influence human comfort and cause sensory effects [17–21]. According to the guideline proposed by Molhave [22], the level of TVOC should not exceed 200 μg m^−3^, in order to ensure human comfort. Concerning ozone, the main health concern is its effect on the respiratory system. Clinical studies have documented an association between short-term exposure to ozone at concentrations of 0.2–0.5 mg m^−3^ (0.4–1 ppm) and mild temporary eye and respiratory irritation and inflammatory response of the respiratory tract and lung [23]. The occupational threshold limit value (TLV) for ozone varies according to the intensity of work from 0.05 to 0.20 ppm (ACGIH TLV 8h-TWA: time-weighted average during an eight-hour workday). Formaldehyde has been classified as a potential carcinogen and can also cause sensory and respiratory irritation [24]. The TLV 8h-TWA according to OSHA is 1 mg m^−3^ (0.75 ppm), while the 15-minute short-term exposure limit (STEL) is 2.5 mg m^−3^ (2 ppm). Carbon dioxide does not cause severe health effects but stands as a significant index of indoor air quality since it is influenced by the number of persons in a room and the rate of air changes.

The aim of this study was to investigate the indoor air environment in four beauty salons located in greater Athens area (Greece).

## 2. Experimental Section

Four randomly selected beauty salons in the greater Athens area (suburbs) were investigated for several parameters. Salons were chosen to be outside the heavily polluted city center in order to avoid the influence of outdoor pollution [25,26]. The salon characteristics are shown in Table 1.

One day measurements during working hours were carried out in each salon in July 2009. The investigation of each salon included one day measurements of chemical and physical parameters as well as inspection of the prevalent conditions. The parameters measured were TVOC, individual volatile organic compounds, ozone, formaldehyde and carbon dioxide. Smoking was not allowed inside the salons.

Particularly, the measurements of the volatile organic compounds were carried out in three salons in the specific area where therapies were performed using products containing volatile organic compounds (body hydrotherapy and care, body massage, reflexology, aromatherapy *etc*.).

VOCs were collected in charcoal sorbent tubes, using sampling pumps (SKC 224PCTX8 and 224PCEX8). The sampling duration was approximately 7–9 hours with an air flow of 0.6 L min^−1^. The analysis of the VOCs collected in the charcoal tubes was performed using Liquid Desorption, Gas Chromatography Mass Spectrometry. The detection limit of the method was about 1 μgm^−3^. All the measurements were area measurements, where the sampler has been placed near the working area of the beautician. More specifically, measurements were performed in rooms where body treatments performed using products containing VOCs.

Short term TVOCs as Toluene were measured using a ppbRAE instrument (equipped with a planar, dual channel PID detector (lamp 10,6 Ev). The sampling duration was approximately 10–15 minutes with an air flow of 0.4 l min^−1^. The measuring scale was 0–200 ppm, with a resolution of 1 ppb. All the measurements were area measurements, where the sampler has been placed near the working area of the beautician.

The measurements of ozone were carried out in four salons, where steam equipment (ozonizer) for facial cleaning was used. An ozonizer, also known as an ozone generator, creates ozone by charging the air with a burst of high negative voltage. It used in cosmetics mainly in facial treatments with ozonated water or steam. Ozone was collected by adsorption in a passive sampling tube (Radiello adsorbing cartridges: RAD 172 adsorbing cartridge, matrix microporous PE tube with 4,4-dipyridylethylene coated silica which was contained in a diffusive body). Ozone was sampled linearly from 10,000 to 4,000,000 μg m^−3^ per minute. The sampling duration was approximately 7–9 hours depending on the working hours of each salon. Desorption was performed with 3-methyl-2-benzothiazolinone hydrazone (MBTH) solution and ozone was determined with visible spectrometry determination of MBTH—azide at 340 nm. The detection limit of the method was about 0.1 μg m^−3^. All the measurements were area measurements, where the sampler has been placed near the ozonizer.

Formaldehyde measurements were performed in three salons during the use of products for nails care. For the detection of formaldehyde, detector tubes (Gastec No 91L) were used along with a Gastec pump (Gastec GV–100). The air passes through chromatometric detector tubes that change color depending on the concentration of the specific parameter (from yellow to reddish brown). The measuring limit was 0.05 ppm.

Carbon dioxide was measured using a TESTO 535 instrument. For the detection of carbon dioxide, TESTO 535 is equipped with a two channel infrared sensor. The measuring scale was 0–9,999 ppm, with a resolution of 1 ppm.

## 3. Results and Discussion

The experimental results regarding the measurements of the TVOCs measured in the salons are given in Table 2. The TVOC levels measured in the beauty salons in 8-hour basis showed a significant variation. More specifically, the concentrations ranged between 100 and 1,450 μg m^−3^. These levels are well below the occupational exposure limits (OELs) established by legislation for individual volatile organic compounds (Table 2). It must be noted however, that these OELs have been developed for a specific compound and industrial areas where workers are usually use appropriate protective equipment (e.g., masks). In addition health and safety requirements should be ensured both for workers and possibly vulnerable customers.

Indoor air TVOCs concentrations showed great variance depending on the use of products and their characteristics, the number of treatments carried out as well as ventilation. Higher concentrations were found in the beauty salon where the maximum number of treatments was performed as it is shown in Table 2. The concentrations of each individual VOC and the corresponding occupational exposure limits as provided by the American Conference of Governmental Industrial Hygienists (ACGIH) are presented in Table 2. VOCs detected in salons varied from area to area depending on the beauty products used. The compounds detected include aromatic compounds (toluene and xylene, isopropyltoluene), as well as ketones and esters usually used as solvents and compounds with characteristic odor such as terpenes.

Toluene and xylenes are derived from many sources such as vehicles (incomplete combustion), solvents used in various products including cosmetic products [25]. The presence of high concentrations of these compounds showed that they are emitted from the products used in those salons. Ethyl acetate and acetone are used as solvents in many products, including nail polish removers. Terpenes such as pinene and limonene give characteristic odor in beauty products. Camphor is a compound with strong odor which when applied on the skin causes cooling sensation. It should be noted that the limit for camphor is much lower than other volatile organic compounds (2 mg m^−3^). Menthenol is a terpene alcohol, which has aromatic properties and can cause irritation to eyes and nose [27]. bis(Trimethylsilyl)salicylate and octamethylcyclotetrasiloxane are used in silicone products. It is worth mentioning that other VOCs were not detected in this survey including benzene, isopropylbenzene, cumene, isopropyl acetate, benzyl acetate, isopentyl alcohol, phenol, and cresol.

The results regarding the short-term measurements of the TVOCs are given in Table 3. In many cases levels were well above 1 mg/m^3^ indicating that employees may be exposed in very high concentrations during treatments, working near product storage area and especially without adequate ventilation.

The results of ozone concentrations in the four salons are presented in Table 4. All the measurements were performed in the room, where the steam equipment (“ozonizer”) for facial cleaning was used. During measurements the steam equipment was used for 1 to 2 hours.

Table 4 shows the number of facial cleanings performed in each salon. The duration of sampling ranged between 5 to 7 hours. Ozone concentrations measured in all salons were quite low since they did not exceed 13.3 μg m^−3^ (Table 4). In all cases the concentrations detected were well below the occupational OSHA threshold limit values (TLV 8h-TWA) of 0.2 mg m^−3^ or the ACGIH TLV 8h-TWA of 0.1 mg m^−3^ for heavy work. This means that the use of steam equipment did not appear to increase ozone levels in the indoor air of the salon.

Formaldehyde measurements were performed in three salons during the use of nail care products. Concentrations detected were lower than the detection limit of the method in all salons (<0.05 ppm) and therefore below the occupational threshold limit values and the osmic level.

Another important factor influencing indoor air quality is carbon dioxide. Carbon dioxide concentrations in the salons are presented in Table 5. Carbon dioxide levels in the indoor air ranged between 402 and 1,268 ppm, depending on the number of persons in the salons during measurements and ventilation. Levels higher than 1,000 ppm, which is the guideline value according to ASHRAE [28] were found in only two cases. Levels above 1,000 ppm (with a density of 0.1 persons m^−2^) show that there were significant ventilation problems.

It should be noted that the levels of carbon dioxide are influenced by the number of persons in each beauty centre during the measurements, which it was small. In general except for one, the salons were small and natural ventilation (opening windows) and small air-conditioning units were available. Due to climate conditions natural ventilation commonly used and as a result when windows were closed the areas were not ventilated properly during the day and especially when treatments were performed to clients. This explains the differences of carbon dioxide levels between salons. It is worth mentioning that in salon 2, in the area for body treatments the concentration found was 1,071 ppm during a body treatment and in the photo—depilation area the concentration detected during photolysis reached 1,268 ppm. As also other studies have shown ventilation is perhaps the most critical factor for achieving high air quality in beauty salons [1–4]. It should be mentioned however that outdoor air pollution may severely influence indoor concentrations of VOCs especially in urban environments, as a previous study in Athens city has shown [25].

## 4. Conclusions

This study examined the exposure of workers in salons to specific pollutants. Four small salons located in the Attica region (Greece) were examined during a typical day. Measurement results showed that:

Volatile organic compounds are the compounds with the greatest interest in beauty salons. The main volatile organic compounds found in the salons were aromatics (toluene, xylene), esters and ketones (ethyl acetate, acetone, *etc*.), odorous terpenes (pinene, limonene, camphor, menthenol) and, camphor. Cosmetologists may be exposed to high concentrations of a mixture of volatile organic compounds at levels that can cause symptoms or discomfort (average daily concentrations reached 1.5 mg m^−3^ in our survey). These levels were influenced by the number of treatments performed, the ventilation, and the number and type of products that were open during the measurements. On the other hand, these levels could be decreased significantly by following certain practices such as good ventilation of the areas, keeping all the beauty products in a separate room, closing the packages of the beauty products after use and finally selecting beauty products without strong odor.

Ozone measurements which were performed in areas where steam equipment (“ozonizer”) for facial cleaning was used showed very low levels (between 0.1 and 13.3 mg m^−3^). It seems that the use of steam equipment is unlikely to increase significantly ozone levels in the indoor air and therefore cosmeticians are unlikely to be exposed to high ozone concentrations. Formaldehyde was not detected in any of the 4 salons during the use of nails products but this finding has to be confirmed in different settings and other products use.

Our findings should be interpreted with caution since chemical exposure conditions in beauty salons are incredibly diverse and variable and any indoor environmental monitoring survey might be far than complete. Symptoms caused by the various compounds include irritation to eyes and nose, headache, dizziness, drowsiness, *etc*. Therefore, the appearance of a mixture of compounds in high concentrations can cause similar symptoms to both employees and customers. It is critical to have further research integrating biological monitoring and workplace assessment, on the possible relation between exposure conditions and subjective symptoms [29]. For exposure control and personal protection against some of the chemical factors, as it is indicated in material safety data sheets (MSDS), it is recommended to ventilate work area, with ten or more air changes per hour. In a previous report in a different setting, we have shown the significance of local exhaust ventilation for personal protection against chemical substances [30]. In addition, respirators with organic carbon chemical cartridges or the N95 respirator dust mask with organic vapor/odor control as a reasonable alternative should be used [28]. This underlines the need to provide information and training in employees about the possible toxic effects of chemicals and education on proper handling, storing and, disposal. But in order to reduce hazards in this work environment the production and use of safer products (less hazardous) might be the first necessity. Since there are not available safety evaluations and occupational exposure limits (OELs) for most of the substances used, the inspection of facilities and enforcement of compliance with existing regulations might be effective in order to ensure normal operating conditions.

## Figures and Tables

**Table 1.** Salons and measurement day characteristics.

	**Salon**			
	**1 [Kifisia]**	**2 [Glyfada]**	**3 [Ano Glyfada]**	**4 [Neo Psychico]**
Personnel	Three	Four	Three	Two
Work (and total) rooms	2 (4)	5 (8)	3 (3)	3 (4)
No of treatment sites per room*				
1^st^ 2^nd^ 3^rd^ 4^th^ 5^th^	2 FT 2 BT; 1 P-D	3 FT 2 P-D 2 BT 2 BT 1 NT	2 FT 2 BT 1 NT; 1 P-D	2 FT 2 BT 1 NT; 1 P-D
Date of sampling	16 July 2009	23 July 2009	23 July 2009	17 July 2009
Services provided this day	4 FT; 2BT; 1P-D	9FT; 3BT; 1NT; 1P-D	4FT; 2BT; 1NT; 1P-D	4FT; 1BT; 1NT
Ventilation **	window & AC unit in each room	window & AC unit in each room	window & AC unit in each room	window & AC unit in each room
Temperature (°C)$	up to 28,2	up to 27,8	up to 28	up to 28
Humidity (%)$	up to 53,8$$	up to 41,3	up to 30,2	up to 53,1$$

* Facial treatment = FT; Body treatment = BT; Photo-depilation = P-D; nail treatment = NT;

** Ventilation: various patterns of open-closed windows and air-conditioning units were monitored throughout the sampling day in every salon;

$ Equipment : Testo 605-H1 according to ISO 7726 (precision 3%RH/0.5°C);

$$ When steam equipment was used.

**Table 2.** Main VOCs found in three salons (8h-TWA levels in μg m^−3^) and the corresponding occupational exposure limits.

	**Salon**			ACGIH (TLV-TWA) mg m^−3^
Compound (μg m^−3^)	**1**	**2**	**3**	
Toluene	3	67	10	187.5
Xylene	n.d.	10	n.d.	435
4-Isopropyltoluene	n.d.	67	n.d.	-
Acetone	n.d.	230	107	1,190
Ethyl acetate	n.d.	23	44	1,400
Butyl acetate	n.d.	n.d.	49	710
Pinene	n.d.	142	6	-
Limonene	n.d.	227	8	-
Eucalyptol	n.d.	48	n.d.	-
Carane	n.d.	n.d.	31	-
Menthenol	n.d.	n.d.	20	-
bis(Trimethylsilyl)salicylate	93	364	n.d.	-
Octamethylcyclotetrasiloxane	n.d.	n.d.	49	120
Camphor	n.d.	115	n.d.	4 (STEL)
**TVOCs as Toluene**	**100**	**1450**	**490**	
Date of sampling	16/7/09	23/7/09	23/7/09	
Duration of sampling	10:00–19:00	10:05–19:15	11:45–18:00	

**ACGIH:** American Conference of Governmental Industrial Hygienists;

**TLV-TWA:** time-weighted average during an eight-hour workday;

**STEL:** short term exposure limit which is the higher permissible exposure level for a period of 15 min.

**Table 3.** Short term (10 min) levels of TVOCs as Toluene in beauty salons.

**Salon**			
**1**	**2**	**3**	**4**
μg/m^3^ time; AC comment	μg/m^3^ time; AC comment	μg/m^3^ time; AC comment	μg/m^3^ time; AC comment
**1180** 10:00; closed Shortly after a BT	**1450** 13:40; closed Shortly after a BT	**1144** 11:50; closed Products storage area	**1320** 12:30; closed Opening FT room
**2980** 10:45; closed Products storage area	**2650** 13:30; closed During a BT	**855** 17:40; open Products storage area	**1790** 12:00; closed During a NT
	**1290** 16:50; open During two FT		
	**2130** 14:40; closed Immediately after a P-D		

AC: air-conditioning; FT: facial treatment; BT: body treatment; P-D: photo-depilation; NT: nail treatment.

**Table 4.** Ozone concentrations measured in four salons.

Salon	Date of sampling	Duration of sampling	Ozone concentration (μg m^−3^)	No of facial cleanings performed (duration of using steam equipment)
1	16/7/09	10:00 – 19:00	13.3	Three (app. 2 hours total).
2	23/7/09	10:00 – 19:15	0.8	Nine (app. 15 minutes for each cleaning).
3	23/7/09	11:45 – 18:00	11.2	Four (app. 2 hours total)
4	17/7/09	12:00 – 19:00	<0.1	Two (app. 1.5 hours total).

**Table 5.** Carbon dioxide concentrations in salons.

**Salon**	**Area of measurement**	**Time of measurement**	**CO_2_ concentration (ppm)**	**Comments**
1	Body treatments	10:00	906	Area had not been ventilated since the previous day
		10:30	710	After ventilation (by opening windows)
	Facial treatments	10:40	645	Area had not been ventilated since the previous day
2	Facial treatments	10:30	567	Windows were closed during the whole day
		14:10	857	
		16:50	948	
	Photo-depilation	10:40	634	Windows were closed during the whole day. This area hadn’t been used before the measurement
		14:30	1,268	Measurement was performed during photo-depilation
	Nails treatments	14:50	723	There is no window in this area
	Body treatments	10:40	748	Windows were closed during the whole day
	2^nd^ area for body treatments	10:25	600	Windows were closed during the whole day
		13:50	1,071	A body therapy was performed and there were 3 persons in the room
3	Body treatments	11:50	523	Windows were open during the whole day
	Facial treatments	12:15	484	
4	Nails treatments	12:00	609	Area hadn’t been ventilated since the previous day
	Facial treatments	12:30	586	Area hadn’t been ventilated since the previous day and there were no one in the room
	Body treatments	13:00	621	Area had not been ventilated since the previous day
